# Role of leisure and sleep in promoting daily physical activity in adults with down syndrome

**DOI:** 10.1080/21642850.2025.2521418

**Published:** 2025-06-22

**Authors:** Melissa R. Jenkins, Victoria L. Fleming, Emily K. Schworer, Yiwen Dong, Dana L. Tudorascu, Benjamin L. Handen, Sigan L. Hartley

**Affiliations:** a Waisman Center, University of Wisconsin–Madison, Madison, Wisconsin, USA; b Department of Human Development and Family Studies, University of Wisconsin–Madison, Madison, Wisconsin, USA; c Department of Biostatistics and Health Data Science, University of Pittsburgh, Pittsburgh, Pennsylvania, USA; d Department of Psychiatry, University of Pittsburgh, Pittsburgh, Pennsylvania, USA

**Keywords:** Down syndrome, leisure, sleep, physical activity, wearable technology

## Abstract

**Background:** Adults with Down syndrome (DS) are at an elevated risk of chronic health conditions (e.g. obesity, Alzheimer’s disease). Although physical activity can mitigate the effects of these conditions, adults with DS spend more time sedentary relative to the adult general population. This study examined how daily fluctuations in two lifestyle factors, sleep and leisure, affect physical activity in adults with DS.

**Method:** For 7 days, a sample of adults with DS (*N* = 109) wore a GT9X ActiGraph accelerometer to collect movement and sleep data and completed a daily diary to record leisure engagement. Movement variables included time spent sedentary, in light physical activity, and in moderate-to-vigorous physical activity. Sleep variables included total sleep time (TST) and sleep efficiency (SE). Time spent in and total count of cognitively stimulating leisure (e.g. reading, experiential activities) and social leisure (e.g. visiting friends, attending club meetings) were based on 22 items from the Victoria Longitudinal Study activity questionnaire. Mixed linear models examined between – and within-person associations for sleep and physical activity, and leisure and physical activity. Models controlled for sociodemographics, day of week, and weekend (vs. weekday).

**Results:** At the between-person level, more TST was associated with more time sedentary (*ps* < .05) and less physical activity (*ps* < .001). More SE was associated with less time sedentary (*ps* < .001) and more physical activity (*ps* < .05). At the within-person level, participants with more physical activity than typical predicted greater TST (and less SE) that night (*ps* < .01). More cognitively stimulating and social leisure were associated with more physical activity at the within-person level (*ps* < .05).

**Conclusions:** Findings can inform health programs seeking to increase physical activity in adults with DS. Interventions should consider the function of sleep and leisure in everyday life for long-term sustainability of physical activity.

More than 250,000 individuals in the United States have Down syndrome (DS), which is caused by the full or partial triplication of chromosome 21. DS is the most common known genetic cause of intellectual disability (Presson et al., 2013) and is associated with a host of co-occurring medical conditions in adulthood such as obesity, cardiovascular conditions, autoimmune conditions, Alzheimer’s disease, and depression and anxiety disorders (Ballard et al., 2016; Glasson et al., 2014; Grieco et al., 2015; Stancliffe et al., 2011). There is a critical need to identify factors that promote behaviors, including physical activity, that promote healthy aging in adults with DS and eliminate or mitigate the effects of co-occurring medical conditions. Relative to the adult general population, adults with DS engage in low levels of physical activity and spend more time in sedentary behaviors such as lying or sitting (Oreskovic et al., 2020; Shields et al., 2018). Among adults with DS, those who are less physically active have worse cognitive performance (Pape et al., 2021), higher body fat and body mass index (BMI) (Boer, 2020; Bühl et al., 2019; Giagkoudaki et al., 2010), and more co-occurring medical health problems (Fleming et al., 2022). Lifestyle factors may play a key role in facilitating or hindering physical activity within the everyday lives of adults with DS. The goal of the proposed study was to examine the connections between daily sleep and leisure activities in promoting or impeding physical activity in adults with DS. This study used self – and informant-reported daily diary indices of leisure activity and actigraphy-derived indices of physical activity and sleep quality to examine the between – and within-person associations between leisure, sleep, and physical activity across a 1-week period in a sample of 109 adults with DS.

Adults with DS experience a high prevalence of sleep problems (Carter et al., 2009; Hoffmire et al., 2014). Across studies, up to 80% of adults with DS experience difficulty falling and staying asleep and 35-78% experience obstructive sleep apnea (OSA) (Giménez et al., 2018; Giménez et al., 2021; Hill, 2016; Santos et al., 2022). These elevated sleep problems could be linked to the reduced physical activity in the DS population, as there are robust associations between sleep and physical activity found in the general population (Kredlow et al., 2015; Zhao et al., 2023). At a global-level, engaging in more physical activity is associated with better quality sleep (e.g. fewer wakes after sleep onset and shorter latency from time in bed to falling asleep) in cross-sectional studies (Brand et al., 2010; Osailan et al., 2021) and longitudinally in exercise intervention trials (Kalak et al., 2012; Nguyen & Kruse, 2012). In the other direction, poor sleep quality (e.g. high number of wakes after sleep onset and difficulty falling and staying sleep) predicts lower future engagement in physical activity at a global level (Haario et al., 2013; Holfeld & Ruthig, 2014). At a daily level, more physical activity during the day has been associated with shorter latency from time in bed to sleep onset and optimal sleep duration that night (Gabriel et al., 2017; Master et al., 2019). Additionally, following a night of higher-rated sleep quality, adults report more physical activity the following day (Dzierzewski et al., 2015; Tang & Sanborn, 2014). Only a handful of studies have examined the effect of sleep on physical activity for adults with DS. In a small sample of adults with DS (*N* = 43), features associated with OSA (e.g. snoring loudly, tongue biting) were related to less engagement in physical activity at a global level based on informant report (Chen & Ringenbach, 2023). Therefore, there is a need to better understand the associations between sleep and physical activity.

Leisure activity has also been seen as a way to build physical activity into everyday life (Orsega-Smith et al., 2007). Leisure activity is generally described as the activities done during one’s free time for enjoyment (i.e. outside of obligatory activities like employment or chores) (Veal, 1992; Wilson, 1980). Common leisure activities reported for adults with DS include watching television, listening to music, reading, and cooking (Carr, 2008; Putnam et al., 1988). In the general population, engaging in more cognitive leisure (e.g. playing games, reading) or social leisure (e.g. visiting friends, attending club meetings) has been reported to be associated with more physical activity, more calories burned, and lower BMI in adults (Pampel, 2012; Pressman et al., 2009), and adolescents (Auhuber et al., 2019; Feldman et al., 2003; Mota et al., 2008). In part, cognitive and social leisure often involve movement and/or physical activity (e.g. bowling with a friend or visiting a friend’s house to play a game). However, cognitive and social leisure may also foster positive mood, promote time management skills, and build friendships in ways that motivate adults with DS to engage in other healthy behaviors including physical activity (e.g. Feldman et al., 2003; Macdonald-Wallis et al., 2012; Rominger et al., 2024). However, little is known about the interrelationships among cognitive and social leisure and physical activity at the daily level. Nothing is known about the role of cognitive and social leisure in fostering physical activity in the everyday lives of adults with DS.

The goal of the present study was to examine the between-person and within-person daily effects of sleep (i.e. total sleep time and sleep efficiency) and cognitive and social leisure (i.e. total time in activities) on daily physical activity (time in sedentary behavior, light physical activity, or moderate-to-vigorous physical activity [MVPA]) across one-week in a sample of 109 adults with DS (aged 25–59). The adult with DS wore a wrist-worn ActiGraph accelerometer to record sleep and physical activity data. Leisure activity was recorded in a daily diary with the help of a study partner. At a between-person level, we hypothesized that adults with DS who had better sleep quality and engaged in more leisure activities would engage in more physical activity. At a within-person level, on days following a better-than-average night of sleep, adults with DS were predicted to engage in more physical activity than was typical for that individual. Similarly, at a within-person level, on days when adults with DS engaged in more leisure activities than average, they were predicted to engage in more physical activity than was typical.

## Methods

### Sample

The current study included 109 adults with DS and a study partner who knew the adult with DS well. The majority of study partners were family members (*n* = 98, 90.7%). Study partners who did not co-reside with the adult with DS had daily contact with them during the course of data collection. Participants were recruited from the Alzheimer Biomarker Consortium-Down syndrome (ABC-DS; Handen et al., 2020), a longitudinal multisite study of the natural history of Alzheimer’s disease, and had agreed to be part of an auxiliary lifestyle study. Eligibility criteria in ABC-DS included: (a) karyotype indicating trisomy 21 (full, partial, or mosaic), (b) 25 years of age or older, and (c) mental age of at least 3 years of age (included because the cognitive battery had only been validated in individuals with mental age of 3 years plus). In the current study, participants were included only if they were deemed to not have dementia based on a clinical case consensus process involving a physician, psychologist, and at least three other study staff who reviewed all available information (i.e. scores from directly-administered cognitive tests, informant-reports of functioning, medical history, recent life events and clinical lab values) (see Handen et al., 2020 for details). In accordance with the Declaration of Helsinki, consent was obtained from the adult with DS or their legal guardian (with assent obtained from the adult with DS).

### Procedures

For 7 consecutive days, the adult with DS and their study partner completed a daily diary in which they jointly recorded information about the adult with DS’s cognitive and social leisure activity. They also recorded information about sleep (e.g. bed and wake time), and physical activity (e.g. time spent exercising) to validate the accelerometer data. During this same 7-day period, the adult with DS wore a wrist-worn GT9X ActiGraph accelerometer to assess physical activity and sleep quality. There were 796 accelerometer observations, of which 749 (94.1%) were usable data; observations were discarded if participants wore the accelerometer for less than 10 daytime hours in a given day (*n* = 47 observations, 5.9%). The average daily accelerometer wear time was 15.02 h (*SD* = 1.86).

### Measures

#### Sociodemographics

Control variables used in analyses were chronological age, intellectual disability level (based on mental age in years), biological sex, and BMI. Intellectual disability level was estimated based on the Stanford Binet Intelligence Test, Fifth Edition (SB5) (Roid, 2003) or the Kaufman Brief Intelligence Test, Second Edition (KBIT-2) (Kaufman & Kaufman, 2004). Intellectual disability level was coded based on mental age as: mild: ≥ 9 years, moderate: 4–8 years, and severe: ≤ 3 years. Mental age was used rather than IQ standard score, given that the lowest possible IQ standardized scores on the SB5 Abbreviated Battery and KBIT-2 are 47 and 40, respectively, which do not allow for the differentiation of moderate and severe/profound intellectual disability level. Biological sex was coded: male = 1 and female = 0. BMI was measured using participants’ weight in kilograms divided by their height in square meters (kg/m^2^).

#### Leisure activity

Leisure activities were reported in the daily diary and jointly completed by the study partner and adult with DS. This method has been found to have good reliability and validity with adults with DS and their families (Esbensen & Hoffman, 2017; Mihaila et al., 2020). A list of activities based on the Victoria Longitudinal Study activity questionnaire (VLS; Jopp & Hertzog, 2007) was used; the VLS has good internal consistency (Jopp & Hertzog, 2010 in general population; Mihaila et al., 2017 in DS population). The VLS includes 68 items across 10 subscales; Jopp and Hertzog (2010) validated the factor structure, yielding distinct domains for social and cognitive leisure utilizing confirmatory factor analyses. For the present study, a smaller list of 22 activities were selected given their likelihood of occurring over the course of a week. Leisure activity items were placed into two domains: cognitive (activities that promote cognitive stimulation; 17 items for experiential, developmental, and game activities) and social (activities that involve social interactions; five items for social-public and social-private activities). See Supplemental Table 1 for activity classification. Two variables were created for cognitive leisure (total count and time in minutes), and two variables were created for social leisure (total count and time in minutes). The time variables were square root transformed to address slight positive skewness. On average, post-transformation skewness for time in cognitive leisure and social leisure was −0.28 and 0.55, respectively.

#### Physical activity

Adults with DS wore a wrist-worn GT9X Actigraph accelerometer (Actigraph LLC, Pensacola, FL) on their non-dominant wrist for a 7-day period. Except during water-based activities, participants were instructed to wear the monitor at all times. The accelerometer sampled at a rate of 30 Hz and epoch length of 60 s. The Troiano algorithm (Troiano et al., 2008) on ActiLife (version 6.13.4) was used to validate wear time. Variables included time (in minutes) spent sedentary, in light physical activity, and in MVPA. Upon examination of the skewness of the data distributions, time sedentary and time in MVPA were square root transformed. On average, post-transformation skewness for time sedentary and in MVPA was 0.40 and 0.23, respectively.

#### Sleep

The GT9X Actigraph accelerometer also recorded sleep data for 7 consecutive nights. Variables included: (1) total sleep time (TST) in minutes and (2) sleep efficiency (SE), defined as the minutes asleep divided by the total number of minutes in bed. The Cole-Kripke algorithm (Cole et al., 1992) was used for sleep-wake identification. The variable SE was reflected in which each value was subtracted from the maximum value (100) plus the constant 1, and then square root transformed to address substantive negative skewness. On average, post-transformation skewness for SE was −0.22.

#### Statistical analysis

Descriptive statistics were used to examine the distribution of study variables, screen for potential outliers, and summarize demographic information and average indices for physical activity (sedentary behavior, light physical activity, and MVPA), sleep (TST and SE), and cognitive and social leisure variables. Mixed linear models were conducted in SAS 9.4 (SAS Institute Inc., Cary, NC) using PROC MIXED to examine between-person and within-person associations between daily physical activity, sleep, and leisure. The focus was on sleep parameters and leisure activity variables as predictors (i.e. independent variables) of physical activity (dependent variable) in models. Thus, primary multilevel models examined the effect of (1) prior night’s sleep on next day physical activity and (2) the effect of same-day leisure activities on physical activity. A secondary model also examined the effect of physical activity on that night’s sleep, given theorized bidirectional associations between sleep and physical activity. All models included random intercepts and a one-day lagged effect controlling for prior day outcomes. For all predictors of interest, fixed effects included within-person (each person’s mean subtracted from their daily value) and between-person (sample mean subtracted from each person’s mean) effects. Between-person fixed effects were also used for covariates included in models: age, intellectual disability level, BMI, and biological sex. Variables for diary day (i.e. first day coded 1, second day coded 2) and weekend day (no coded 0, yes coded 1) were also included.

## Results

Participants had a mean age of 39.83 years (*SD* = 8.80) with a range of 25 and 59 years. Most were white and non-Hispanic (*n* = 97, 89.8%) and more than half were male (*n* = 63, 58.3%). Most participants had either mild (*n* = 48, 44.9%) or moderate intellectual disability (*n* = 49, 45.8%). The majority of participants (*n* = 101) had the karyotype of full trisomy 21. A minority of participants had translocation (*n* = 5) or mosaicism (*n* = 1), or other partial trisomy 21 (*n* = 1). Three-quarters (*n* = 81) of participants lived with family, and 84.2% (*n* = 85) had no or minimum supervision. Table 1 provides additional participant sociodemographic information.

**Table 1.** Demographics and average counts and time (hours) in physical activity, sleep, and leisure engagement.

Variable	Frequency (*N*) or *M*	Percent (%) or *SD*	Range
Sex			
Male	63	58.33	–
Female	45	41.67	–
Race/Ethnicity			
White, non-Hispanic	97	89.81	–
Non-White and/or Hispanic	11	10.19	–
Intellectual Disability			
Mild	48	44.86	–
Moderate	49	45.79	–
Severe	10	9.35	–
BMI	32.05	7.36	18.87–56.90
Age (years)	39.83	8.80	25–59
Residence			
Family Caregiver	81	75.00	–
Group Home	12	11.11	–
Independent Living	15	13.89	–
Level of Independence			
Minimum Supervision	74	73.27	–
24 h Supervision	16	15.84	–
No Supervision	11	10.89	–
Physical Activity			
Time Sedentary	3.35	2.17	0–12.95
Time Light Activity	9.18	1.98	2.31–15.02
Time MVPA	2.57	1.88	0–11.02
Sleep			
Total Sleep Time	6.62	1.62	1.37–12.32
Sleep Efficiency	71.75	14.32	6.09 –100
Social Leisure			
Activity Count	1.42	1.09	0–5
Time	2.37	3.03	0–21
Cognitively Stimulating Leisure			
Activity Count	3.64	2.17	0–10
Time	4.22	3.03	0–19

Note. BMI = body mass index. MVPA = moderate-to-vigorous physical activity.

### Sleep predicting physical activity

Table 2 presents the between-person and within-person effects of prior-night sleep on physical activity. At the between-person level, more TST was associated with more time spent sedentary (*b* = 0.01, *p* = .02, 95% CI [0.00, 0.02]). Less TST significantly predicted more time spent in light physical activity (*b* = −0.39, *p* < .001, 95% CI [−0.61, −0.17]) and MVPA (*b* = −0.02, *p* < .001, 95% CI [−0.02, −0.01]). Participants with less SE spent more time sedentary (*b* = −1.28, *p* < .001, 95% CI [−1.92, −0.64]). More SE was positively associated with light physical activity (*b* = 19.62, *p* = .02, 95% CI [3.10, 36.14]) and MVPA (*b* = 0.83, *p* = .01, 95% CI [0.16, 1.49]). Weekend day was associated with physical activity in all models; participants engaged in more sedentary behavior and less light physical activity and MVPA on the weekend as compared to weekdays. Age significantly predicted time in light activity (*b* = 3.65, *p* < .001, 95% CI [1.56, 5.75]) and time in MVPA (*b* = −0.11, *p* < .01, 95% CI [−0.20, −0.03]) in the TST predictor models. Age significantly predicted time in light activity (*b* = 3.45, *p* < .01, 95% CI [1.30, 5.61]) and time in MVPA (*b* = −0.12, *p* < .01, 95% CI [−0.21, −0.03]) in the SE predictor models. Biological sex was a predictor of time in MVPA (*b* = −1.57, *p* = .05, 95% CI [−3.13, −0.01]) in the SE predictor model.

**Table 2.** Sleep predicting next day physical activity.

	Sedentary Time (minutes)				Light Time (minutes)				MVPA Time (minutes)			
Predictor	B	95% CI	SE	t	B	95% CI	SE	t	B	95% CI	SE	t
Total Sleep Time (minutes)												
Intercept	14.10	[13.26, 14.95]	0.43	32.80***	573.12	[551.33, 594.91]	11.06	51.81***	12.00	[11.20, 12.81]	0.41	29.51***
Between	0.01	[0.00, 0.02]	0.00	2.33*	−0.39	[−0.61, −0.17]	0.11	−3.50***	−0.02	[−0.02, −0.01]	0.00	−3.56***
Within	−0.00	[−0.01, 0.00]	0.00	−2.05*	−0.36	[−0.46, −0.26]	0.05	−6.98***	−0.00	[−0.01, 0.00]	0.00	−1.49
Age	−0.01	[−0.10, 0.08]	0.04	−0.17	3.65	[1.56, 5.75]	1.06	3.46***	−0.11	[−0.20, −0.03]	0.04	−2.66**
Sex	0.70	[−0.86, 2.25]	0.78	0.89	0.12	[−36.95, 37.19]	18.69	0.01	−1.43	[−2.92, 0.06]	0.75	−1.90
ID	−0.15	[−1.31, 1.01]	0.59	−0.25	1.23	[−26.55, 29.02]	14.01	0.09	−0.85	[−1.96, 0.27]	0.56	−1.51
BMI	−0.02	[−0.12, 0.08]	0.05	−0.40	0.86	[−1.51, 3.23]	1.19	0.72	0.01	[−0.09, 0.10]	0.05	0.12
Lag PA	−0.18	[−0.27, −0.08]	0.05	−3.70***	−0.02	[−0.11, 0.07]	0.04	−0.43	−0.04	[−0.13, 0.05]	0.04	−0.93
Sleep Efficiency (percentage)												
Intercept	14.16	[13.34, 14.98]	0.42	34.06***	577.15	[554.56, 599.74]	11.47	50.32***	12.03	[11.21, 12.85]	0.42	28.96***
Between	−1.28	[−1.92, −0.64]	0.32	−3.97***	19.62	[3.10, 36.14]	8.33	2.36*	0.83	[0.16, 1.49]	0.33	2.48*
Within	−0.14	[−0.37, 0.09]	0.12	−1.18	0.68	[−6.54, 7.90]	3.68	0.19	−0.07	[−0.28, 0.14]	0.11	−0.63
Age	−0.01	[−0.09, 0.07]	0.04	−0.26	3.45	[1.30, 5.61]	1.09	3.18**	−0.12	[−0.21, −0.03]	0.04	−2.73**
Sex	1.09	[−0.42, 2.59]	0.76	1.43	−3.32	[−42.08, 35.44]	19.54	−0.17	−1.57	[−3.13, −0.01]	0.78	−2.00*
ID	0.00	[−1.09, 1.11]	0.55	0.01	−6.26	[−34.61, 22.09]	14.29	−0.44	−1.12	[−2.26, 0.01]	0.57	−1.96
BMI	−0.05	[−0.14, 0.05]	0.05	−0.98	1.46	[−0.99, 3.92]	1.24	1.18	0.03	[−0.07, 0.13]	0.05	0.58
Lag PA	−0.16	[−0.25, −0.07]	0.05	−3.38***	−0.02	[−0.11, 0.08]	0.05	−0.32	−0.05	[−0.14, 0.04]	0.04	−1.09

Note. *N* = 107. Unstandardized beta coefficients reported with standard errors. MVPA = moderate-to-vigorous physical activity. ID = intellectual disability. BMI = body mass index. PA = physical activity. Lag PA is one-day lagged effect of time spent sedentary, in light physical activity, and MVPA, respectively.

*p < .05. **p < .01. ***p < .001.

At the within-person level, following a night with lower-than-average TST, participants spent more time in sedentary behavior (*b* = −0.00, *p* = .04, 95% CI [−0.01, 0.00]). Additionally, following a night with lower-than-average TST, participants engaged in more light physical activity (*b* = −0.36, *p* < .001, 95% CI [−0.46, −0.26]). There were no significant within-person effects of prior night SE on physical activity.

### Physical activity predicting sleep

Table 3 presents between-person and within-person effects of physical activity predicting sleep that night. At the between-person level, participants who engaged in more sedentary behavior on average had more TST (*b* *=* 5.77, *p* < .01, 95% CI [1.87, 9.68]); in this model, weekend days also predicted more TST (*b* *=* 12.17, *p* = .04, 95% CI [0.51, 23.82]). More time spent in light physical activity (*b* *=* −0.31, *p* < .001, 95% CI [−0.46, −0.15]) and MVPA (*b* *=* −7.08, *p* < .001, 95% CI [−10.93, −3.23]) was associated with less TST. On average, participants who were more sedentary had less SE (*b* = −0.11, *p* < .001, 95% CI [−0.16, −0.06]). Additionally, participants who engaged in light physical activity (*b* = 0.00, *p* = .01, 95% CI [0.00, 0.01]) and MVPA (*b* = 0.06, *p* = .03, 95% CI [0.01, 0.12]) throughout the week had more SE. Biological sex significantly predicted SE in the sedentary (*b* = 0.47, *p* = .03, 95% CI [0.04, 0.89]) and MVPA (*b* = 0.48, *p* = .04, 95% CI [0.03, 0.93]) predictor models. Age significantly predicted TST (*b* = 2.07, *p* = .03, 95% CI [0.26, 3.87]) in the light physical activity predictor model.

**Table 3.** Physical activity predicting same night’s sleep.

	Total Sleep Time (minutes)				Sleep Efficiency (percentage)			
Predictor	B	95% CI	SE	t	B	95% CI	SE	t
Sedentary Time (minutes)								
Intercept	384.52	[365.81, 403.24]	9.50	40.46***	5.24	[4.98, 5.50]	0.13	39.09***
Between	5.77	[1.87, 9.68]	1.97	2.93**	−0.11	[−0.16, −0.06]	0.03	−4.15***
Within	−9.18	[−13.81, −4.54]	2.35	−3.90***	0.15	[0.08, 0.21]	0.03	4.47***
Age	1.00	[−0.77, 2.76]	0.89	1.12	−0.01	[−0.03, 0.02]	0.01	−0.53
Sex	−15.69	[−47.14, 15.77]	15.86	−0.99	0.47	[0.04, 0.89]	0.21	2.18*
ID	20.91	[−2.41, 44.23]	11.76	1.78	−0.03	[−0.35, 0.29]	0.16	−0.18
BMI	−0.58	[−2.59, 1.42]	1.01	−0.58	−0.02	[−0.04, 0.01]	0.01	−1.24
Lag Sleep	−0.20	[−0.28, −0.10]	0.04	−4.26***	−0.14	[−0.23, −0.06]	0.04	−3.25**
Light Time (minutes)								
Intercept	385.90	[367.49, 404.31]	9.35	41.27***	5.25	[4.98, 5.52]	0.14	38.20***
Between	−0.31	[−0.46, −0.15]	0.08	−3.95***	0.00	[0.00, 0.01]	0.00	2.49*
Within	0.25	[0.08, 0.42]	0.09	2.87**	−0.00	[−0.01, −0.00]	0.00	−2.96**
Age	2.07	[0.26, 3.87]	0.91	2.27*	−0.02	[−0.04, 0.01]	0.01	−1.23
Sex	−11.37	[−41.94, 19.19]	15.41	−0.74	0.39	[−0.06, 0.83]	0.22	1.73
ID	20.02	[−2.72, 42.77]	11.47	1.75	−0.03	[−0.36, 0.31]	0.17	−0.18
BMI	−0.44	[−2.40, 1.52]	0.99	−0.44	−0.02	[−0.05, 0.01]	0.01	−1.20
Lag Sleep	−0.20	[−0.30, −0.11]	0.05	−4.28***	−0.15	[−0.24, −0.07]	0.04	−3.46***
MVPA (minutes)								
Intercept	385.63	[367.15, 404.12]	9.39	41.09***	5.24	[4.97, 5.51]	0.14	38.00***
Between	−7.08	[−10.93, −3.23]	1.94	−3.65***	0.06	[0.01, 0.12]	0.03	2.17*
Within	9.53	[4.90, 14.15]	2.34	4.06***	−0.10	[−0.16, −0.03]	0.03	−2.80**
Age	0.17	[−1.62, 1.97]	0.91	0.19	0.00	[−0.03, 0.03]	0.01	0.04
Sex	−21.55	[−52.75, 9.64]	15.72	−1.37	0.48	[0.03, 0.93]	0.23	2.10*
ID	13.61	[−9.73, 36.95]	11.77	1.16	0.02	[−0.32, 0.36]	0.17	0.14
BMI	−0.57	[−2.53, 1.40]	0.99	−0.57	−0.02	[−0.04, 0.01]	0.01	−1.13
Lag Sleep	−0.18	[−0.27, −0.09]	0.05	−3.98***	−0.15	[−0.24, −0.07]	0.04	−3.49***

Note. *N* = 109. Unstandardized beta coefficients reported with standard errors. ID = intellectual disability. BMI = body mass index. MVPA = moderate-to-vigorous physical activity. Lag Sleep is one-day lagged effect of total sleep time and sleep efficiency, respectively.

*p < .05. **p < .01. ***p < .001.

At the within-person level, on days when participants were more sedentary than average (*b* = −9.18, *p* < .001, 95% CI [−13.81, −4.54]), they had less TST that night. On days that participants engaged in more light physical activity (*b* = 0.25, < .01, 95% CI [0.08, 0.42]) and MVPA (*b* = 9.53, *p* < .001, 95% CI [4.90, 14.15]) than average, they had more TST that night. On days that participants were more sedentary, they had more SE that night (*b* *=* 0.15, *p* < .001, 95% CI [0.08, 0.21]). Finally, on days that participants engaged in less light physical activity (*b* *=* −0.00, *p* < .01, 95% CI [−0.01, −0.00]) and MVPA (*b* *=* −0.10, *p* < .01, 95% CI [−0.16, −0.03]), they had higher SE that night.

### Leisure predicting physical activity

Table 4 presents the between-person and within-person effects of leisure predicting same-day physical activity. There were no significant between-person effects of cognitively stimulating or social leisure on same-day physical activity. Similar to the sleep models, participants engaged in more sedentary behavior on weekend days versus weekdays, and age significantly predicted more time in light activity and less time in MVPA in all models. At the within-person level, on days when a participant spent more than their average time in social leisure, they also spent significantly more time in light physical activity (*b* = 1.55, *p* < .01, 95% CI [0.40, 2.70]). Additionally, total count of cognitively stimulating leisure (*b* *=* 5.10, *p* = .04, 95% CI [0.27, 9.94]) was associated with light physical activity while time spent in cognitively stimulating leisure (*b* *=* 0.07, *p* < .01, 95% CI [0.03, 0.11]) was associated with MVPA.

**Table 4.** Leisure predicting same day physical activity.

	Sedentary Time (minutes)				Light Time (minutes)				MVPA Time (minutes)			
Predictor	B	95% CI	SE	t	B	95% CI	SE	t	B	95% CI	SE	t
Any Social (count)												
Intercept	14.29	[13.42, 15.15]	0.44	32.50***	578.61	[555.84, 601.37]	11.56	50.06***	11.95	[11.01, 12.79]	0.43	28.01***
Between	−0.41	[−1.35, 0.53]	0.47	−0.86	22.76	[−0.30, 45.83]	11.63	1.96	0.24	[−0.70, 1.19]	0.48	0.51
Within	−0.17	[−0.46, 0.12]	0.15	−1.17	8.21	[−0.44, 16.86]	4.40	1.87	0.09	[−0.16, 0.34]	0.13	0.72
Age	−0.01	[−0.10, 0.09]	0.05	−0.14	3.79	[1.53, 6.06]	1.14	3.32**	−0.11	[−0.20, −0.02]	0.05	−2.44*
Sex	0.65	[−0.94, 2.25]	0.80	0.82	−0.78	[−39.71, 38.14]	19.62	−0.04	−1.22	[−2.82, 0.38]	0.81	−1.51
ID	−0.05	[−1.26, 1.15]	0.61	−0.09	−0.75	[−30.31, 28.81]	14.91	−0.05	−0.98	[−2.14, 0.27]	0.61	−1.60
BMI	−0.03	[−0.13, 0.07]	0.05	−0.59	1.40	[−1.03, 3.84]	1.23	1.14	0.00	[−0.10, 0.10]	0.05	0.00
Lag PA	−0.16	[−0.26, −0.06]	0.05	−3.15**	−0.01	[−0.10, 0.08]	0.05	−0.23	−0.05	[−0.14, 0.04]	0.04	−1.16
Social Time (minutes)												
Intercept	14.27	[13.41, 15.13]	0.44	32.71***	578.23	[555.37, 601.08]	11.60	49.84***	11.96	[11.12, 12.81]	0.43	28.10***
Between	−0.13	[−0.28, 0.02]	0.08	−1.73	2.75	[−0.99, 6.49]	1.89	1.46	0.08	[−0.07, 0.23]	0.08	1.03
Within	−0.01	[−0.05, 0.03]	0.02	−0.50	1.55	[0.40, 2.70]	0.58	2.65**	0.00	[−0.03, 0.03]	0.02	0.06
Age	−0.01	[−0.09, 0.08]	0.04	−0.12	3.36	[1.15, 5.58]	1.11	3.02**	−0.12	[−0.21, −0.03]	0.05	−2.55*
Sex	0.71	[−0.86, 2.28]	0.79	0.90	0.48	[−38.72, 39.67]	19.76	0.02	−1.26	[−2.84, 0.33]	0.80	−1.57
ID	−0.05	[−1.21, 1.11]	0.59	−0.08	−5.35	[−34.41, 23.71]	14.65	−0.37	−0.98	[−2.16, 0.20]	0.59	−1.65
BMI	−0.03	[−0.13, 0.07]	0.05	−0.64	1.44	[−1.02, 3.90]	1.24	1.16	0.00	[−0.10, 0.10]	0.05	0.03
Lag PA	−0.16	[−0.26, −0.06]	0.05	−3.18**	−0.01	[−0.10, 0.08]	0.05	−0.26	−0.05	[−0.14, 0.04]	0.04	−1.14
Any Cognitively Stimulating (count)												
Intercept	14.27	[13.42, 15.13]	0.43	32.88***	578.30	[555.54, 601.06]	11.56	50.04***	11.96	[11.12, 12.80]	0.42	28.20***
Between	−0.39	[−0.80, 0.01]	0.20	−1.93	9.75	[−0.38, 19.88]	5.11	1.91	0.26	[−0.15, 0.67]	0.21	1.24
Within	−0.15	[−0.31, 0.01]	0.08	−1.84	5.10	[0.27, 9.94]	2.46	2.07*	0.08	[−0.06, 0.22]	0.07	1.10
Age	−0.00	[−0.09, 0.09]	0.04	−0.05	3.35	[1.17, 5.54]	1.10	3.04**	−0.12	[−0.21, −0.03]	0.04	−2.61*
Sex	0.42	[−1.14, 1.98]	0.79	0.53	6.89	[−32.04, 45.83]	19.63	0.35	−1.07	[−2.65, 0.51]	0.80	−1.34
ID	0.03	[−1.11, 1.18]	0.58	0.06	−7.28	[−35.93, 21.36]	14.44	−0.50	−1.03	[−2.19, 0.14]	0.59	−1.75
BMI	−0.02	[−0.12, 0.07]	0.05	−0.48	1.27	[−1.18, 3.71]	1.23	1.03	−0.00	[−0.10, 0.10]	0.05	−0.07
Lag PA	−0.16	[−0.26, −0.06]	0.05	−3.20**	−0.01	[−0.10, 0.08]	0.05	−0.18	−0.05	[−0.14, 0.04]	0.04	−1.07
Cognitively Stimulating Time (minutes)												
Intercept	14.28	[13.41, 15.15]	0.44	32.42***	578.10	[555.04, 601.17]	11.71	49.37***	12.00	[11.16, 12.84]	0.42	28.27***
Between	−0.03	[−0.19, 0.12]	0.08	−0.46	1.38	[−2.38, 5.15]	1.90	0.73	0.10	[−0.05, 0.25]	0.08	1.29
Within	−0.05	[−0.10, 0.00]	0.03	−1.83	−0.04	[−1.55, 1.47]	0.77	−0.05	0.07	[0.03, 0.11]	0.02	3.11**
Age	0.00	[−0.09, 0.09]	0.04	0.08	3.24	[1.02, 5.46]	1.12	2.90**	−0.12	[−0.21, −0.03]	0.04	−2.61*
Sex	0.52	[−1.07, 2.11]	0.80	0.65	4.79	[−34.78, 44.37]	19.95	0.24	−1.04	[−2.63, 0.54]	0.80	−1.30
ID	0.11	[−1.06, 1.28]	0.59	0.19	−9.54	[−38.72, 19.65]	14.72	−0.65	−1.14	[−2.30, 0.03]	0.59	−1.93
BMI	−0.03	[−0.13, 0.07]	0.05	−0.54	1.29	[−1.20, 3.78]	1.25	1.03	−0.01	[−0.11, 0.09]	0.05	−0.11
Lag PA	−0.16	[−0.26, −0.06]	0.05	−3.22**	−0.01	[−0.11, 0.08]	0.05	−0.31	−0.05	[−0.13, 0.04]	0.04	−1.03

Note. *N* = 108. Unstandardized beta coefficients reported with standard errors. ID = intellectual disability. BMI = body mass index. MVPA = moderate-to-vigorous physical activity. Lag PA is one-day lagged effect of time spent sedentary, in light physical activity, and MVPA, respectively.

*p < .05. **p < .01. ***p < .001.

## Discussion

The purpose of this study was to examine how sleep and leisure affect physical activity in the everyday lives of adults with DS. A key finding is the connection, at both a global and daily level, between physical activity and sleep. At the between-person level, adults with DS who engaged in more light physical activity on average had lower TST and higher SE than those who engaged in less physical activity across the 7 days. At the within-person level, on days when an adult with DS had more TST than typical, they engaged in less next-day physical activity. In turn, on days when adults with DS engaged in more physical activity than typical, they had greater TST (and less SE) than typical that night. This could be a result of the energy expended during physical activity and the need for restorative sleep, as well as thermoregulation (elevated body temperature during movement and subsequent lower body temperature during sleep) (Best et al., 2019; Buman & King, 2010). These results align with the meta-analytic findings from general population samples and indicate that engaging in more physical activity (relative to others and relative to one’s own typical pattern) is associated with less TST and more SE (Atoui et al., 2021).

The sociodemographic variables were related to lifestyle differences. In our sample, older (versus younger) adults with DS engaged in more light physical activity and less MVPA, which is consistent with prior research on the general population (Bélanger et al., 2011), and may be driven by aging-related decreases in motor planning, coordination, and balance (Smith & Ulrich, 2008). In our sample, men (versus women) had more efficient sleep (i.e. higher SE), on average, which aligns with findings in the general population (Madrid-Valero et al., 2017). Prior DS research has not reported biological sex differences when examining OSA indices (Giménez et al., 2021); thus, future research is needed to understand the nuanced ways that sleep may vary by biological sex in DS. Lifestyle activities did not vary based on intellectual disability level or BMI. Some prior studies have reported that adults with severe (versus mild or moderate) intellectual disability engage in less physical activity (Vancampfort et al., 2022) and have more sleep problems (Harvey et al., 2003), although others have not (Gawlik et al., 2017; Luiselli et al., 2005). Our study primarily included adults with DS with mild to moderate intellectual level, and was underpowered to identify any differences for adults with DS with severe or profound intellectual level. In prior work, higher BMI has also been associated with poor sleep, in part due to increased risk for OSA (Jehan et al., 2017; Punjabi, 2008). Although there was a moderate correlation between OSA and BMI in our study (*r* = .323, *p* < .001), OSA was not a significant predictor of physical activity and sleep in sensitivity analyses. BMI effects in and of itself may be reduced in DS given structural differences associated with trisomy 21 (e.g. narrowed airway and low muscle tone).

At a within-person level, on days when an adult with DS engaged in more than typical social and cognitive leisure, they also had more than typical physical activity. These findings suggest that often cognitive and social leisure activity may have beneficial effects for healthy aging because they involve physical activity (e.g. going bowling with a friend involves light to moderate physical activity). Additionally, cognitive and social leisure have been linked to positive mood in ways that motivate adults with DS to engage in physical activity (e.g. Pressman et al., 2009; Rominger et al., 2024).

### Strengths and limitations

A strength of this study was the use of objective measures of sleep and physical activity; prior studies in the DS population have typically relied on subjective measures, such as caregiver reports. Additionally, the multilevel (i.e. inter – and intra-individual effects) and bidirectional analyses conducted have been underutilized in samples of adults with DS. The sample size in the current study is also larger than what is commonly featured in accelerometer studies (Ngueleu et al., 2022; Wu et al., 2023), increasing the rigor of findings. There were several study limitations. The adult with DS and their study partner were instructed to jointly record leisure each day. Most study partners co-resided with the adult with DS and others had daily contact. However, it is possible that leisure activities were inaccurately reported. Furthermore, the daily diary did not inquire about substance use (e.g. alcohol, caffeine) which can impact sleep (e.g. SE and TST) (Clark & Landolt, 2017; Feige et al., 2006). While multilevel effects across 7 days and the inclusion of a one-day lagged effect were incorporated into models, Atoui et al. (2021) recommends that daily diary studies analyze data for minimally 12 consecutive days to properly examine different lagged associations. Finally, it is possible that some of the leisure activities designated as cognitive leisure also involved social interactions and vice versa.

### Implications and future directions

Given that the majority of adults with DS in this study resided with family caregivers, it is important to understand how lifestyle factors and/or associations between sleep, leisure, and physical activity may alter for adults with DS living independently or in other settings. It is also important to understand how these associations may change with age as Alzheimer’s disease symptomology unfolds, as the adults with DS in the present study were non-demented. On the earlier side of the life course, results from this study can inform approaches to sleep health for children with DS, primarily in investigating everyday contributors or effects beyond physical activity such as learning and cognition. Future research should also examine if findings on the role of sleep and leisure in promoting physical activity extend to adults with other types of intellectual and developmental disabilities and thus can extend policy and intervention recommendations more broadly to the intellectual and developmental disability community. Future studies should also extend the daily diary and actigraphy accelerometer collection to one month to afford more sophisticated time series analysis for better understanding temporal ordering of effects among sleep, leisure, and physical activity at a within-person level (Irish et al., 2014).

## Conclusion

Leisure activities and sleep have important associations with physical activity in the everyday life of adults with DS. These findings have relevance for interventions aimed at increasing physical activity. Specifically, findings suggest that efforts to improve sleep in adults with DS (e.g. increasing screening efforts and improving treatment options for addressing sleep disruptions and sleep disordered breathing problems) and efforts to increase social and cognitive leisure activities in everyday life may play an important role in promoting more physical activity. Comprehensive health interventions – i.e. those that target multiple lifestyle domains (sleep, leisure and physical activity) – may lead to the most sustained benefits given the interconnections among lifestyle domains in the lives of adults with DS.

## Data Availability

Data for this study was obtained from The National Institutes of Health (NIH) National Institute on Aging (NIA) Alzheimer’s Biomarkers Consortium – Down Syndrome (ABC-DS), the following licenses/restrictions apply: Applicants must complete and submit a data request form (ABC-DS Data Request Form) and review and sign the ABC-DS Data Use Agreement. Requests to access these datasets should be directed to ABC-DS, https://www.nia.nih.gov/research/abc-ds#data
